# Neuron–Microglia Interactions in Mental Health Disorders: “For Better, and For Worse”

**DOI:** 10.3389/fimmu.2016.00544

**Published:** 2016-11-29

**Authors:** Eric S. Wohleb

**Affiliations:** ^1^Department of Psychiatry, Yale University School of Medicine, New Haven, CT, USA; ^2^Department of Psychiatry and Behavioral Neuroscience, University of Cincinnati College of Medicine, Cincinnati, OH, USA

**Keywords:** neuroplasticity, microglia, anxiety, depression, post-traumatic stress disorder, neuroinflammation, parainflammation, neuroimmune

## Abstract

Persistent cognitive and behavioral symptoms that characterize many mental health disorders arise from impaired neuroplasticity in several key corticolimbic brain regions. Recent evidence suggests that reciprocal neuron–microglia interactions shape neuroplasticity during physiological conditions, implicating microglia in the neurobiology of mental health disorders. Neuron–microglia interactions are modulated by several molecular and cellular pathways, and dysregulation of these pathways often have neurobiological consequences, including aberrant neuronal responses and microglia activation. Impaired neuron-microglia interactions are implicated in mental health disorders because rodent stress models lead to concomitant neuronal dystrophy and alterations in microglia morphology and function. In this context, functional changes in microglia may be indicative of an immune state termed parainflammation in which tissue-resident macrophages (i.e., microglia) respond to malfunctioning cells by initiating modest inflammation in an attempt to restore homeostasis. Thus, aberrant neuronal activity and release of damage-associated signals during repeated stress exposure may contribute to functional changes in microglia and resultant parainflammation. Furthermore, accumulating evidence shows that uncoupling neuron–microglia interactions may contribute to altered neuroplasticity and associated anxiety- or depressive-like behaviors. Additional work shows that microglia have varied phenotypes in specific brain regions, which may underlie divergent neuroplasticity observed in corticolimbic structures following stress exposure. These findings indicate that neuron–microglia interactions are critical mediators of the interface between adaptive, homeostatic neuronal function and the neurobiology of mental health disorders.

## Introduction

Mental health disorders, such as anxiety and depression, are a major source of disability leading to significant social and economic burden throughout the world (1–3). While progress has been made in understanding the neurobiology of mental health disorders, it is clear that multiple subtypes exist with varied pathophysiological mechanisms (4). Clinical and preclinical studies show that anxiety- and depressive-like symptoms are linked to altered neuroplasticity in corticolimbic brain regions. In particular, divergent responses are reported; neuronal atrophy and synapse loss in the prefrontal cortex and hippocampus, and neuronal hypertrophy and increased synaptic density in the amygdala and nucleus accumbens (5). Moreover, clinical studies show that many anxiety and depressive symptoms develop or worsen following exposure to psychosocial and environmental stress (6–8). This is pertinent as rodent models of stress can provide insight into the mechanisms that contribute to the neurobiology of anxiety- and depressive-like behaviors. Indeed several stress models, including repeated social defeat and chronic unpredictable stress, recapitulate key neuroplasticity changes that contribute to susceptibility and development of anxiety- and depressive-like behaviors (9–11).

Coinciding with altered neuroplasticity, repeated stress exposure leads to dysregulation of neuroimmune systems that are implicated in mental health disorders (12–15). Microglia are tissue-resident macrophages in the brain that integrate stress-induced neuroimmune signals leading to behavioral consequences (16). Following stress exposure, microglia undergo dynamic alterations in morphology and function within corticolimbic brain regions implicated in anxiety- and depressive-like symptoms (17). In line with these findings, recent studies demonstrate that microglia have an integral role in shaping neuronal responses (i.e., activity) and synaptic elements (i.e., dendrites and dendritic spines) (18), which support adaptive behavior and cognition (19). Thus, stress-associated changes in neuron–microglia interactions may play an integral role in the pathophysiology of mental health disorders. Clinical studies using histological analyses suggest that microglia have altered morphology and function in depressed individuals (20, 21). Moreover, a clinical neuroimaging study showed that individuals experiencing a major depressive episode have enhanced positron emission topography (PET) labeling of the translocator protein (TSPO), a putative marker of neuroinflammation and microglia activation (22). Further studies using microarray gene expression analyses demonstrate increased cytokine and complement pathways in the PFC and hippocampus of postmortem samples obtained from depressed individuals (23, 24). In contrast, clinical reports examining cerebrospinal fluid showed no changes in immune-related markers in depressed patients (25, 26), suggesting that neuroimmune dysregulation may represent a pathophysiological mechanism in a subset of depressed patients. Notably, clinical data showed that individuals with atypical depression had higher levels of inflammatory markers in circulation compared to controls and those with melancholic depression (27). Altogether, clinical studies and preclinical models provide evidence that neuroimmune alterations provoked by stressors may contribute to the neurobiology of mental health disorders in a subset of individuals.

Concomitant neuronal dystrophy and microglia activation implicate reciprocal neuron-microglia interactions in behavioral deficits, and these responses may not directly lead to neuroinflammation *per se*. As researchers demonstrate the ever-changing form and functions of microglia, it is clear that morphological characteristics may reflect several phenotypes. Thus, microglia appear to display specialized responses that are brain region-dependent and dictated by the stress model and duration. These brain region-specific microglia phenotypes may play a role in divergent neuroplasticity observed in corticolimbic brain regions following stress exposure (28). It is also important to note that stress-induced neuroimmune alterations are modest compared to other pathological situations (29). These neuroimmune processes mediated by microglia do not generally lead to neurotoxicity but may contribute to neuronal dystrophy following stress. In this context, stress-associated functional changes in microglia may be indicative of an immune response similar to parainflammation (30), rather than “neuroinflammation.” This notion of stress-induced parainflammation in the brain will be discussed further in this review. Here, literature are presented showing that neuron–microglia interactions have an integral role in promoting homeostasis in the brain as well as how perturbations in neuron–microglia interactions lead to impaired neuroplasticity. In particular, this review will focus on evidence suggesting that repeated stress exposure leads to dysregulated neuron–microglia interactions and neuroplasticity deficits with implications for mental health and neurological disorders.

## Neurons Regulate Microglia Function

**Figure 1:**
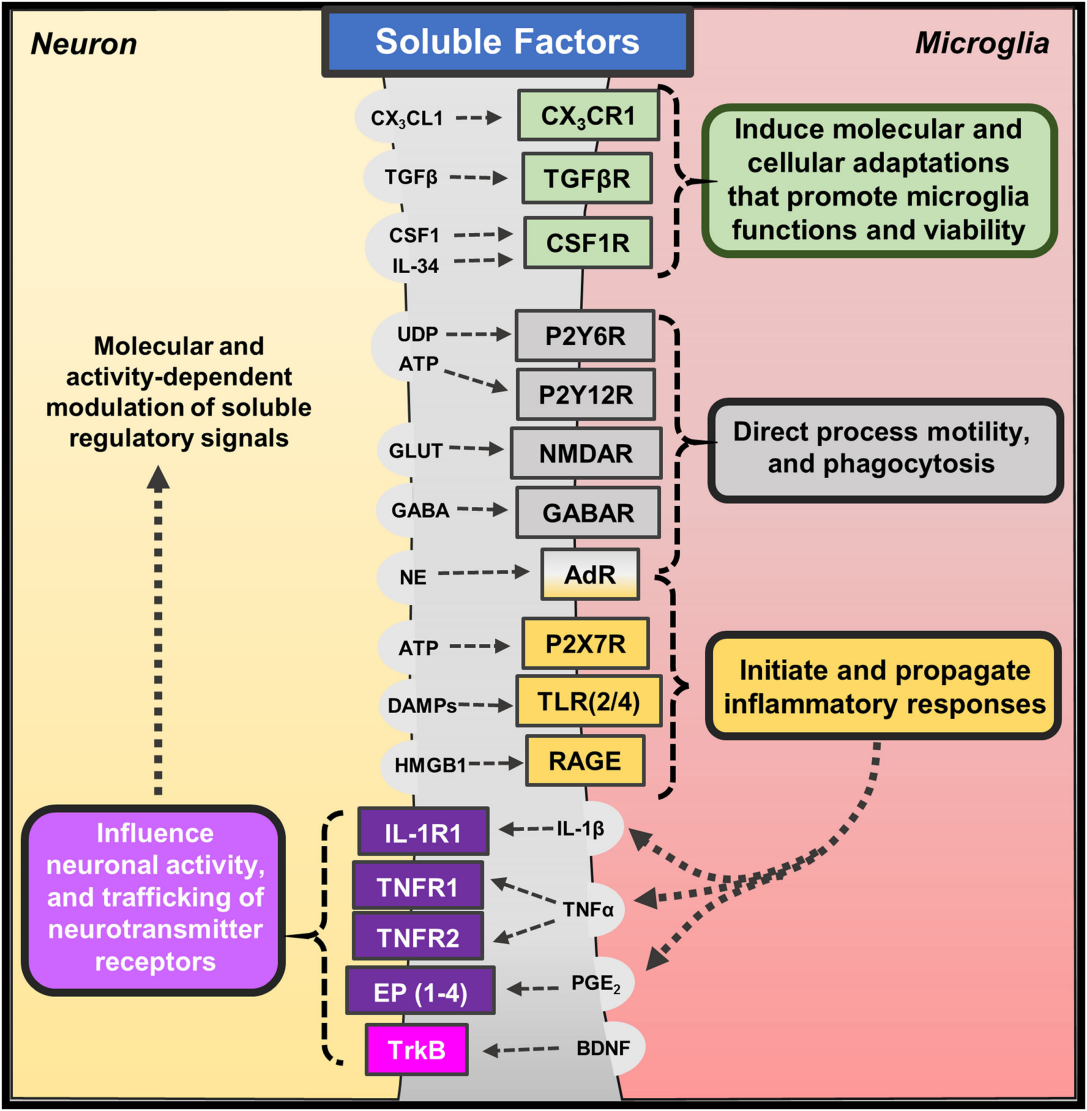
**Soluble factors regulating neuron–microglia interactions**. Several molecular and cellular pathways mediate neuron–microglia interactions. Neurons release soluble immune-related factors (fractalkine – CX_3_CL1; transforming growth factor-β – TGFβ; colony-stimulating factor-1 – CSF1; interleukin-34 – IL-34), nucleotides (uridine diphosphate – UDP; adenosine triphosphate – ATP), neurotransmitters (glutamate – GLUT; γ-aminobutyric acid – GABA; norepinephrine – NE), and danger- or damage-associated molecules (DAMPs; high mobility group box 1 – HMGB1) that bind to cognate receptors on microglia. These soluble neuron-derived signals promote many homeostatic functions but can also initiate or propagate neuroinflammation depending on the context. Microglia also release soluble factors, such as cytokines (interleukin-1β – IL-1β; tumor necrosis factor-α – TNFα), prostaglandins (prostaglandin E_2_ – PGE_2_), and neurotrophins (brain-derived neurotrophic factor – BDNF), which bind to neuronal receptors. Microglia often release these factors in response to fluctuations in neuron-derived signals; yet, low levels of microglia-derived cytokines are shown to promote homeostatic neuroplasticity through trafficking of neurotransmitter receptors.

The mammalian central nervous system develops circuits of interconnected neurons that underlie complex functions, including cognition and behavior (31–33). Several cell types residing in the brain contribute to the development and maintenance of this neurocircuitry. Important cellular counterpart of neurons are microglia, which are brain-resident macrophages that direct homeostatic functions and mediate immune responses to pathological conditions (13, 34). Microglia are distributed throughout the brain but appear to have varied roles in specific regions and develop unique features based on tissue-specific molecular signals (28, 35, 36). Recent studies reveal that microglia are maintained in the brain through self-renewal (37, 38), suggesting that these long-lived brain-resident macrophages maintain long-term interactions with proximal neurons. Neuron–microglia interactions are mediated by soluble factors as well as contact-dependent mechanisms (39, 40), and reciprocal communication is necessary for adaptive neuroplasticity and behavior. These features place microglia as a critical mediator of neuronal function for better, and for worse (Figure 1).

Neurons regulate microglia function through soluble factors, including chemokines, cytokines, and neurotransmitters (Figure 1). Neuron-derived fractalkine (CX_3_CL1) regulates microglia activation through binding to CX_3_CR1, which is enriched on microglia (41). CX_3_CL1 is expressed by neurons in two forms (membrane-bound or cleaved soluble proteins) that may transduce different molecular signals (42). The CX_3_CL1–CX_3_CR1 pathway is shown to be critical for proper neurodevelopment, and the absence of this pathway significantly impaired brain connectivity leading to social interaction deficits (43–45). In addition, recent work shows that microglia develop a unique phenotype based on soluble molecular cues likely derived from proximal neurons. For instance, transforming growth factor (TGF)-β promotes a unique transcriptional profile that organizes transcriptional pathways within microglia (35, 36). Other studies show that colony-stimulating factor (CSF)-1 and interleukin (IL)-34 signal microglia through CSF1 receptor to regulate their development and viability (46). Indeed, microglia do not develop in mice lacking CSF1 receptor (47), and recent work shows pharmacological blockade of CSF1 receptor caused depletion of microglia in the brain (37). These studies highlight some of the soluble neuron-derived signals that shape microglia morphology and function.

The distinct, surveying phenotype of microglia allows them to rapidly respond to perturbations in their microenvironment along with deviations in neuronal homeostasis and activity (48). These responses are mediated, in part, by neuronal release of nucleotides (i.e., UDP, ATP) (49) and neurotransmitters [i.e., glutamate, GABA, and norepinephrine (NE)] (19). Indeed, acute glutamate uncaging induced microglia chemotaxis and convergence of microglia processes toward sites of increased neuronal activity (50). Other studies show that microglia morphology and process motility is influenced by extracellular glutamate, GABA, and NE (51). The chemotactic properties of ATP appear to be predominantly mediated by microglia expression of P2Y12 receptor. For instance, microglia lacking P2Y12 receptors display similar baseline surveillance of regional microenvironments; however, their motility is impaired during injury responses when nucleotides are released (52). Similar reports suggest that fluctuations in calcium signaling along with ATP signaling through P2Y12 receptors lead to microglia process convergence as well (53). Interestingly, a recent study indicated that microglia expression of P2Y12 receptors is required for proper development of visual cortex and ocular dominance (54). Other purinergic receptors, such as P2Y6, may play a functional role in these responses as UDP signaling through P2Y6 receptors increased microglia-mediated phagocytosis following hippocampal excitotoxicity (55). These findings indicate that nucleotides released following neuronal activity act as attractants for microglia processes, and dysregulated neuroplasticity is observed in the absence of these interactions.

## Microglia Direct and Shape Neuronal Function

Microglia release soluble factors, including cytokines and prostaglandins, which reciprocally influence and modulate neuronal function (Figure 1). There are significant primary research reports and reviews that describe how cytokines and other immune mediators released by microglia influence neuronal function and neuroplasticity. Indeed, low levels of IL-1β are required for long-term potentiation, while basal levels of TNFα are necessary for proper homeostatic trafficking of AMPA and GABA_A_ receptors, termed synaptic scaling (56, 57) [see also reviews, Ref. (58, 59)]. Based on these studies, it is evident that microglia-mediated cytokine and prostaglandin synthesis can modulate neuronal responses during physiological and pathological conditions. Further studies indicate that microglia-derived cytokines can indirectly affect neurons through gliotransmission mediated by astrocytes (60). For instance, the release of TNFα by activated microglia potentiated astrocyte glutamate release, which can modulate synaptic plasticity and even lead to neurotoxicity (61). In addition, ATP released by microglia is shown to induce glutamate release by astrocytes thereby acutely exciting proximal neurons (62). These indirect signaling pathways may be further augmented during inflammatory conditions. For example, microglia activation and TNFα release increased neuronal hyperactivity and susceptibility to develop seizures (63). Other data show that microglia can support adaptive synaptic plasticity through the release of neurotrophic factors, such as brain-derived neurotrophic factors (BDNF) (64). These findings are not entirely surprising because microglia as the tissue-resident macrophages of the brain mediate pathological processes along with reparative or growth responses (65).

**Figure 2:**
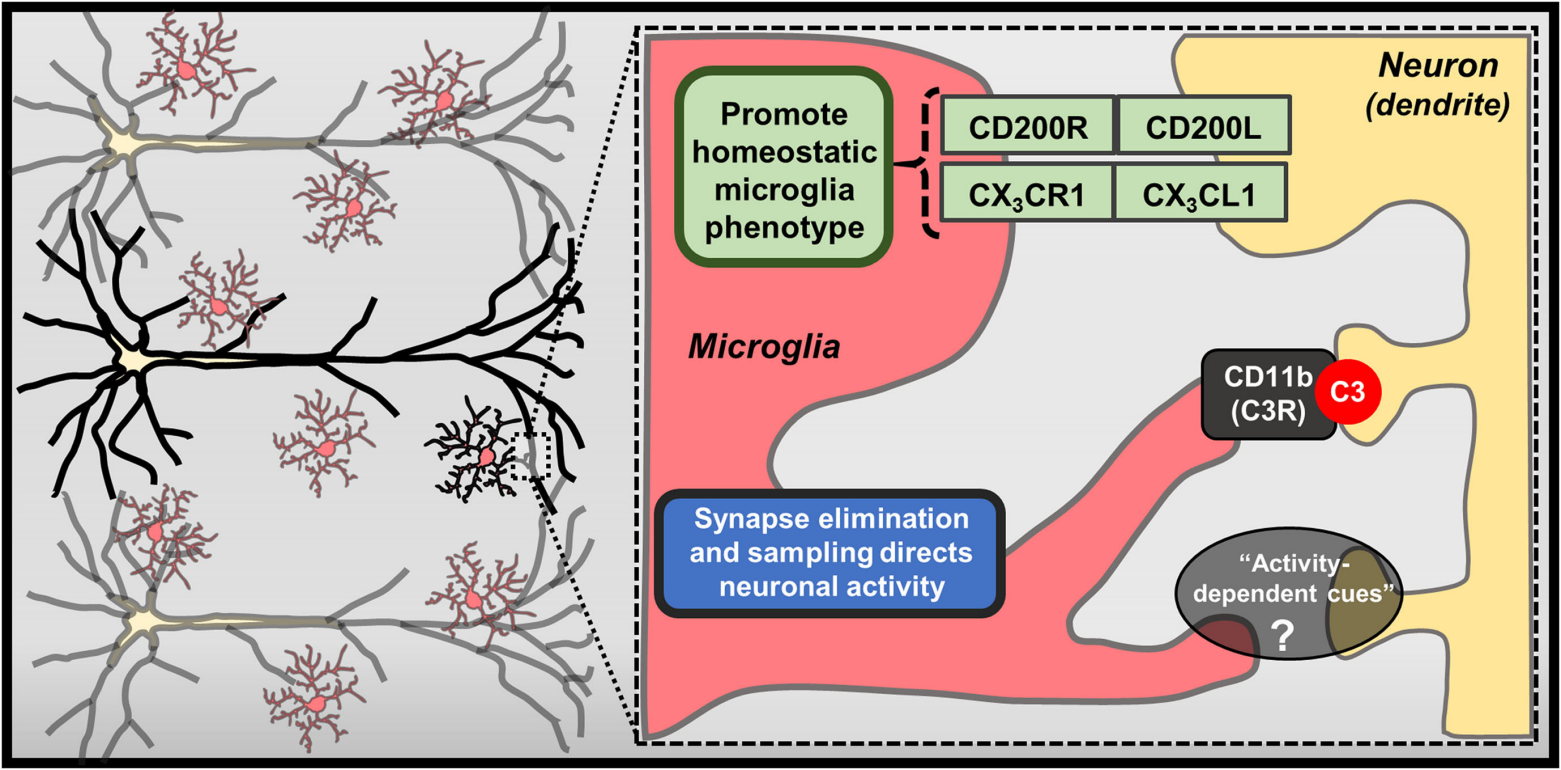
**Contact-dependent mechanisms mediating neuron–microglia interactions**. Physical interactions between neurons and microglia exist, including membrane-bound CX_3_CL1 and CD200L, which bind CX_3_CR1 and CD200R on microglia, respectively. Further recent studies have highlighted specialized mechanisms that contribute to contact-dependent synaptic modulation, such as binding of complement component C3 to synapses and eventual removal of these “tagged” synapses by microglia through CD11b/CR3-mediated phagocytosis. Microglia can also envelop hyperactive neurons and regularly perform “synapse sampling” *via* activity-dependent mechanisms that are not entirely defined (?).

Recent studies also reveal that microglia direct neuronal function through contact-dependent mechanisms, including engulfment of synaptic and dendritic elements (18, 19) (Figure 2). Seminal studies showed that microglia actively phagocytose synapses during neurodevelopment (66). Synapse elimination during neurodevelopment is mediated by complement factor 3 and 1b, which bind synapses with diminished activity, initiating microglia-mediated phagocytosis *via* complement receptor 3 (CR3; CD11b) (67). Other formative studies showed that microglia perform regular, but brief “synapse sampling” by contacting synapses in the adult brain. Of note, *in vivo* imaging showed that microglia sampled proximal synapses once per hour and were drawn more frequently to active synapses. Moreover, these studies show that prolonged microglia interactions resulted in synapse loss (68). Other studies show similar activity-dependent microglia-mediated synapse elimination occurs in cortical brain regions (69). In these studies, soluble factors that modulate neuron–microglia interactions may play a prominent role as purinergic signaling along with release of neurotransmitters that rapidly draw microglia processes toward elevated neuronal activity. For instance, glutamate uncaging caused attraction of microglia processes, which subsequently surrounded hyperactive neurons, leading to contact-dependent reductions in neuronal activity (50). While it is clear that microglia can physically remove synapses, it remains to be determined what mechanisms contribute to microglia-mediated reductions in neuronal activity (Figure 2). These findings demonstrate that microglia are directed by soluble neuron-derived cues to initiate contact-dependent regulation of neuronal activity.

To further examine the functional role of microglia in various physiological and pathological conditions, several groups have developed methods to deplete microglia (70). Initial studies provide compelling evidence that microglia are necessary for adaptive neuroplasticity and behavior. For instance, mice treated with clodronate liposomes showed robust microglia depletion in the hippocampus, which led to spatial memory decrements as well as reduced sociability. These cognitive and social deficits were recapitulated with widespread microglia depletion using the CSF1 receptor antagonist PLX3397. Of note, cognitive and behavioral consequences of microglia depletion were attenuated following repopulation (71). Other studies revealed that clodronate depletion of microglia resulted in enhanced synapses and excitatory input on hippocampal neurons (72). These neurobiological effects have functional implications as pharmacogenetic microglia depletion caused impairments in the rotarod motor learning task (64). These deficits in motor learning were recapitulated when BDNF expression was selectively deleted from microglia. Despite these findings, other studies indicate that widespread microglia depletion with PLX3397 caused no significant alterations in cognition or behavior (37). The dynamic function of microglia in these depletion studies likely reflects their compartmentalized brain region-specific functions (28, 73, 74). These unique neuron–microglia interactions highlight the complexity of molecular and cellular pathways that regulate neurobiology and behavior.

In the end, reciprocal neuron–microglia interactions are regulated by soluble and contact-dependent pathways. These pathways enable microglia to obtain feedback on neuronal functions and rapidly enact interventions to maintain tissue homeostasis. These neuron–microglia interactions appear to support neuronal homeostasis because perturbations in these pathways often result in neuroplasticity impairments and influence performance in memory-based tasks (Figures 1 and 2). In this context, neuron–microglia interactions may be disrupted during pathological conditions, such as mental health and neurological diseases. Further studies will be reviewed to provide evidence that neuron–microglia interactions may play a critical role in the neurobiology of mental health disorders.

## Psychosocial and Environmental Stressors Cause Concomitant Neuronal Dystrophy and Microglia Activation

**Figure 3:**
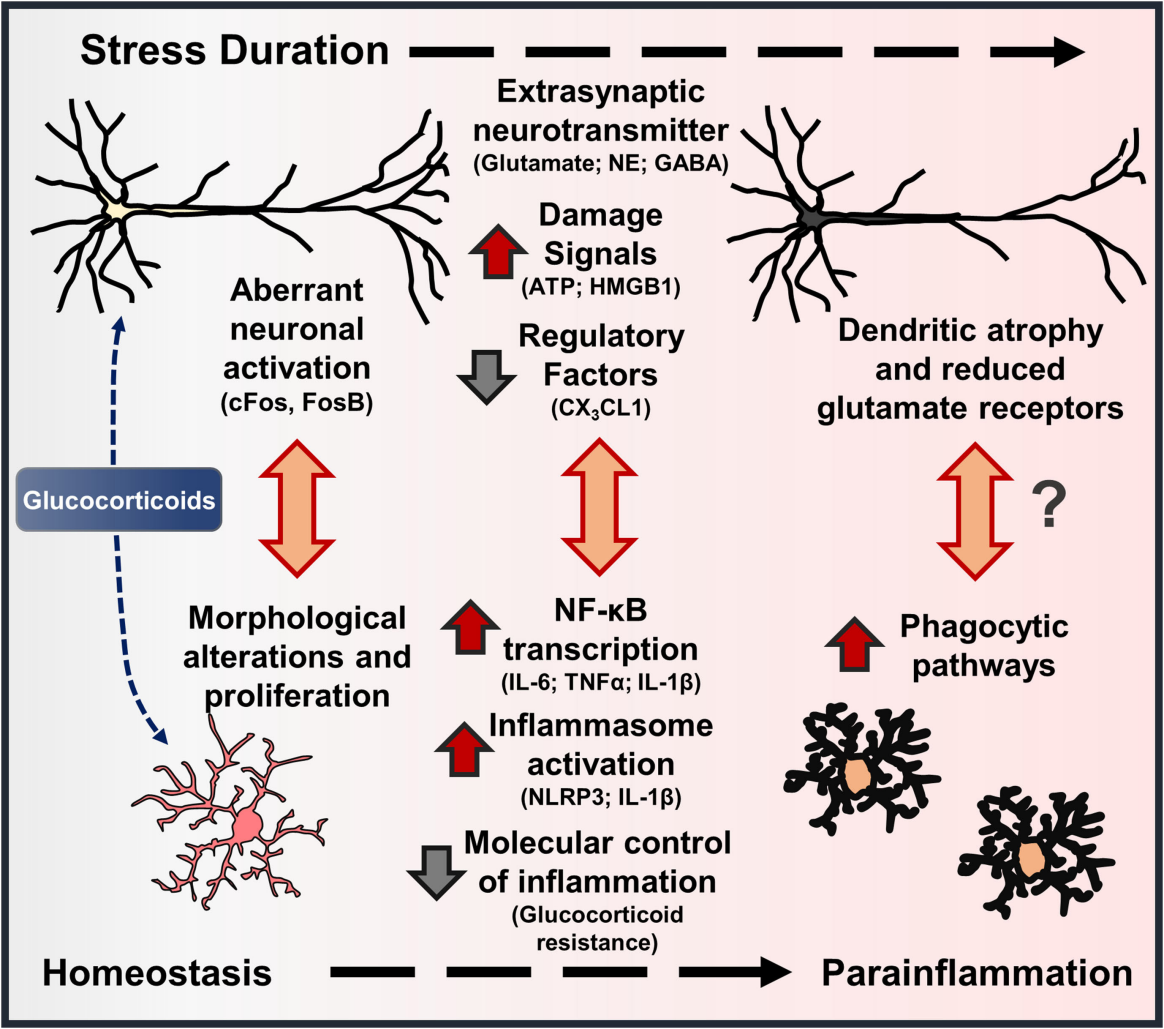
**Stress-induced neuronal dystrophy contributes to alterations in microglia function and parainflammation**. Models of environmental and psychosocial stress activate characteristic neuroendocrine (i.e., glucocorticoids – GC) and neuroimmune pathways that contribute to neuroplasticity alterations underlying anxiety- and depressive-like behaviors. In this cascade, there are features that show stress-induced “neuroinflammation” resembles parainflammation. As brain-resident macrophages, microglia interact with proximal neurons, and disruptions in neuron–microglia interactions initiate morphological and functional changes in microglia. Repeated stress (i.e., prolonged elevations in glucocorticoids) leads to aberrant neuronal activation, extrasynaptic neurotransmitter levels, and release of neuron-derived damage signals. These signals further promote mild to moderate levels of neuroinflammatory transcription and inflammasome activation in microglia. With persistent or chronic stress, pyramidal neurons undergo dendritic atrophy and reduce glutamate receptors, which may be precipitated by cytokines released by proximal microglia or microglia-mediated synapse elimination. Collectively, these stress-induced neuronal responses and resultant molecular and cellular cascades resemble a state of parainflammation.

Exposure to psychosocial and environmental stress is shown to cause robust neuronal activation (i.e., cFos, FosB) through the release of glutamate and NE in corticolimbic brain regions, such as the prefrontal cortex, amygdala, and hippocampus (75–78). Further perceived perturbations of homeostasis caused by stress lead to neuroendocrine activation and release of glucocorticoids (GC) into circulation (79). Converging lines of evidence indicate that aberrant neuronal activation coupled with elevated GC levels lead to neuronal dystrophy in corticolimbic brain regions following stress (Figure 3). For instance, repeated stress caused dendritic atrophy and synapse loss on pyramidal neurons in the rat prefrontal cortex (80, 81), and these effects were recapitulated with chronic GC administration (82). Stress-induced atrophy of pyramidal neurons may be related to impaired glutamate receptor (i.e., NMDA, AMPA) expression in the prefrontal cortex, which is dependent on GC receptor activation (83). Recent work also suggests that dysregulation of interneurons may contribute to disrupted microcircuitry in the prefrontal cortex and depressive-like behavior (84, 85). Together, these neurobiological alterations contribute to shifted excitatory–inhibitory tone in the prefrontal cortex, which is proposed to underlie anxiety and depressive symptoms (9, 86, 87) (Figure 3). Further, brain regions that receive PFC projections, including amygdala, hypothalamus, hippocampus, and nucleus accumbens, exhibit disrupted function as well (88, 89).

Corresponding with altered neuroplasticity in these corticolimbic regions, several reports demonstrate changes in microglia morphology in overlapping brain regions following stress (Figure 3). Indeed, morphological changes in microglia are observed in the prefrontal cortex, amygdala, hypothalamus, hippocampus, and nucleus accumbens following stress exposure (16, 17, 90). Several neuronal and endocrine pathways are shown to cause microglia activation in rodent models of stress. For instance, early work showed that noradrenergic pathways in the brain contribute to elevations in stress-induced pro-inflammatory cytokine expression (91, 92). Further work showed that blockade of β-adrenergic receptors with propranolol prevented microglia activation and pro-inflammatory cytokine gene expression in enriched microglia following repeated social defeat (93). Using inescapable footshock stress, Frank et al. showed that RU486 can prevent stress-induced microglia priming in the hippocampus, suggesting that GCs contribute to these neuroimmune responses as well (94–96). Similar studies using repeated restraint stress showed that pharmacological blockade of NMDA receptors or GC receptors can attenuate stress-associated microglia proliferation, providing evidence that microglia responses are caused by aberrant neuronal activation and neuroendocrine responses (97). In contrast to these findings, recent work by Yirmiya and colleagues showed that chronic stress exposure caused microglia to undergo atrophy in the dentate gyrus of the hippocampus, and these deficits were associated with depressive-like behaviors (98). Further peripheral administration of agents that stimulate microglia function (i.e., LPS, CSF1) reversed microglia atrophy and provided antidepressant effects (98). These compelling findings suggest that brain region-specific signals lead to microglia dysfunction, which can have deleterious effects on neurobiology and behavior as well. Collectively, these studies indicate that dynamic, brain region-specific functional changes in microglia are driven by stress-induced neuroendocrine and neurotransmitter pathways.

Stress-induced microglia activation may also stem from increased release of danger-associated molecular patterns (DAMPs) that bind pattern recognition receptors (PRR) to promote neuroinflammatory signaling through activation of the NLRP3 inflammasome (99). For instance, stress-induced release of the DAMP, high mobility box group (HMGB)-1, caused microglia priming through inflammasome activation and increased pro-inflammatory gene expression (100). In other studies, repeated stress is shown to increase tissue IL-1β levels and depressive-like behaviors; these neuroimmune and behavioral responses are prevented in mice lacking the NLRP3 inflammasome (101, 102). This is consistent with recent work that showed during immobilization stress there is influx of extracellular glutamate and ATP in the hippocampus, followed by elevations in IL-1β and TNFα. Of note, increased release of IL-1β and TNFα is blocked by administration of P2X7 receptor antagonist. In addition, follow-up studies showed that stress-induced depressive-like behaviors were blocked by the P2X7 receptor antagonist and in mice lacking the NLRP3 inflammasome (103). Other reports suggest that increased NLRP3 activation and IL-1β levels in the prefrontal cortex are mediated by microglia following chronic stress, and these responses are reversed with chronic fluoxetine treatment (104). These studies support previous work showing that chronic stress promoted elevations in IL-1β signaling, which contributed to reduced hippocampal neurogenesis and depressive-like behavior (105, 106). Another recent study showed that restraint stress increased TNFα levels in the hippocampus with delayed elevations of several inflammatory mediators in the amygdala, but not in the prefrontal cortex (107). As noted, neuron-derived CX_3_CL1 acts as integral regulator of microglia function, and deficient signaling can result in neuroplasticity deficits (45). Thus, stress-induced reductions in CX_3_CL1 may contribute further to microglia activation and anxiety- or depressive-like behaviors (108). These studies provide evidence that fluctuations in neuron-derived signals (i.e., ATP, HMGB1, and CX_3_CL1) act to regulate microglia activation and subsequent production of pro-inflammatory cytokines with repeated stress exposure, which will influence neuronal function and behavior.

In line with these findings, recent studies show that anxiolytic and antidepressant treatments can block or reverse stress-induced microglia activation. For instance, administration of imipramine following repeated social defeat reduced IL-6 expression in enriched microglia and attenuated depressive-like behaviors following (109). This is consistent with other studies showing selective serotonin reuptake inhibitors produce anti-inflammatory responses in microglia (110). Further work using repeated social defeat demonstrated that pretreatment with benzodiazepines reduced markers of neuroinflammation and diminished anxiety-like behavior (109). Similarly, pretreatment with the non-selective β-adrenergic receptor antagonist propranolol prevented microglia activation and blocked the development of anxiety-like behavior following repeated social defeat (93). These findings raise the question whether these interventions that targeted neurotransmitter systems subsequently attenuated neuroimmune activation or if microglia were the primary effector. In the end, these results suggest that pharmacological treatments may produce anxiolytic or antidepressant effects by simultaneously normalizing neurotransmission and modulating microglia functions. Furthermore, these findings highlight the need to determine cell type-specific pathways that lead to neuronal dystrophy or microglia activation, respectively. As an example, stress-induced microglia activation can be prevented by GC blockade; however, this may be an effect of limiting neuronal dystrophy and diminished release of damage-associated factors or direct effects on microglia. Dissociating the molecular and cellular pathways that contribute to neuronal and microglia adaptations following repeated stress exposure may show novel pharmacological or molecular therapeutic targets.

## Stress-Induced “Neuroinflammation” Resembles Parainflammation

Based on the overlap of stress-induced neuronal dystrophy and functional changes in microglia, it is evident that stress-induced “neuroinflammation” may reflect an inflammatory state termed parainflammation (30). The notion of parainflammation is related to the condition originally described as “physiological inflammation” by Élie Metchnikoff (111). Indeed, parainflammation is an immune state induced by “stressed or malfunctioning” tissues and is considered to be an intermediate phase between homeostasis and classical inflammation. Moreover, parainflammation appears to be mediated predominantly by tissue-resident macrophages, such as microglia. Thus, microglia activation following stress exposure and reported in depression may represent a state of parainflammation, in part, as a response to neuronal dystrophy. In support of this idea, it is evident that stress-induced microglia production of pro-inflammatory cytokines is modest compared to other pathological conditions (29). Moreover, aberrant neuronal activation and damage signals released during stress exposure appear to provoke microglia activation (99), which contributes to altered neuroplasticity and anxiety- or depressive-like behaviors. Stress duration and intensity likely influence the scale of parainflammation, and these prolonged impairments in neuron–microglia interactions may lead to irreversible neurobiological consequences and mental health disorders (Figure 2).

It is worthwhile to note that parainflammation is not considered an entirely detrimental state because it is provoked by malfunctioning cells, and the objective is to restore tissue homeostasis (112). There is evidence that microglia-mediated activation or “neuroinflammation” in non-pathological conditions may initiate adaptive functions to restore neuronal activity to basal, physiological levels. For instance, repeated peripheral endotoxin challenge caused microglia processes to interfere with inhibitory interneuron synaptic connections on pyramidal neurons in the cortex, which provided neuroprotection through increased neuronal activity (113). In separate studies microglia were shown to play a neuroprotective role during excitotoxic injury, with microglia depletion leading to enhanced susceptibility to neuronal death (114). Consistent with these studies, recent work showed that microglia processes contacted swollen axonal segments during excitotoxic conditions, which normalized the excitability of affected neurons and preserved their viability (115). In these instances, neuron–microglia interactions are critical to maintain homeostasis in the brain but likely lead to undesirable inflammatory consequences. It is critical to point out that in many pathological conditions inflammatory responses are required to limit neuronal death and promote tissue repair processes (65, 116). In this context, stress-induced parainflammation and alterations in microglia function may be aimed to restore neuronal homeostasis. It is possible that these processes are initially protective but may generate pathological consequences with chronic stress exposure. Further studies will need to be performed to establish the dynamic role of microglia in these neurobiological responses.

This is not a novel proposal as recent reviews have proposed that a subset of depressed individuals develop a chronic state of parainflammation that contributes to the pathophysiology underlying their symptoms (4, 117). Others have suggested similar models in which stress exposure causes neuronal microdamage, and neuroinflammatory responses are initiated by microglia to promote repair (118). Depending on the neurocircuitry affected, it is argued that anxiety- or depressive-like symptoms may develop, suggesting that neuroinflammatory pathways may lead to divergent neurobiological changes (118). The neuronal microdamage model is compelling; however, further studies will need to be performed to determine molecular and cellular mediators of these effects. The specific molecular and cellular mediators of stress-induced parainflammation are particularly relevant in the context of potential therapies for mental health disorders. For instance, some common antidepressant drugs, such as selective serotonin reuptake inhibitors, have reported anti-inflammatory effects (119). In other cases, the antidepressant behavioral effects of serotonin reuptake inhibitors were blocked by anti-inflammatory drugs (120). These data demonstrate that dynamic neuron–microglia interactions modulate behavior and suggest that parainflammation may enact microglia-mediated mechanisms that normalize neuronal function. In either case, further studies will need to examine the neuron–microglia interactions that contribute to stress-induced parainflammation, and how interventions can engage these mechanisms to provide therapeutic benefits.

## Role of Peripheral Bone Marrow-Derived Myeloid Cells in Stress-Induced Parainflammation

Another important mediator of neuroimmune functions are peripheral immune cells, which propagate immune signals in the brain and influence behavior. Indeed, studies indicate that specific behavioral issues, such as pathological grooming, can be attributed to peripheral hematopoietic immune cells (121). In the context of stress, recent evidence demonstrates a role of peripheral myeloid-derived cells (monocytes and granulocytes) in “neuroinflammation” and anxiety- or depressive-like behavior (14, 90). In these studies, social defeat increased monocyte recruitment to the brain through canonical chemokine pathways (CCL2–CCR2 and CX_3_CL1–CX_3_CR1) (108). Moreover, peripheral monocytes may be preferentially recruited to specific brain regions through adhesion molecule expression (122). In the context of neuroinflammation, it is plausible that this is an attempt to restore homeostasis as neuronal stress-associated signals reach upper limits of parainflammation (112). Initial studies show that stress caused peripheral monocytes to infiltrate the brain under defined conditions; however, further work revealed that peripheral monocytes do not significantly contribute to brain-resident microglia populations (123, 124). Further, pharmacological or genetic techniques to limit monocyte trafficking in the brain and neuroinflammation prevent social defeat-induced anxiety-like behaviors (14, 90). Together, these studies revealed that peripheral monocytes reinforce neuroinflammatory processes and highlight a novel neuroimmune axis that promotes mood disturbances (90, 125). Further studies will need to be performed using microglia- or monocyte-specific genetic techniques or depletion to distinguish cell type-specific contributions to reported neuroimmune mechanisms and their influence on neuronal responses.

## Brain Region-Specific Microglia Responses may Underlie Divergent Neuroplasticity Observed in Models of Stress-Induced Mental Health Disorders

Microglia are implicated in the neurobiology of several mental health disorders; however, it is unclear how they contribute to divergent neuroplasticity alterations in corticolimbic brain regions. It is plausible that brain region-specific neuron–microglia interactions contribute to divergent neuroplasticity reported. For instance, work by Hinwood and colleagues showed that the putative microglia inhibitor minocycline attenuated alterations in microglia morphology and reduced FosB activation in the medial prefrontal cortex, and prevented working memory deficits following repeated stress exposure (126). These results suggest that microglia activation may modulate neuronal responses and cognitive deficits in a rodent stress model. While there is limited clinical evidence showing microglia modulation of neurons, a recent study showed that elevated C-reactive protein levels in circulation, indicative of low grade peripheral inflammation, is associated with increased glutamate levels in the basal ganglia of depressed patients (127). As noted, microglia may modulate neurobiological and behavioral consequences of psychological stress through the release of pro-inflammatory cytokines (i.e., IL-1β, TNFα, and IL-6) (13, 14, 16). Indeed pro-inflammatory cytokines released by microglia following repeated stress exposure may produce brain region-specific alterations in synaptic plasticity. For instance, TNFα administration on pyramidal neurons derived from the hippocampus increased AMPA receptor trafficking to postsynaptic sites and concomitant reductions in GABA_A_ receptors that caused increased excitability (57, 128). In contrast, TNFα administration on striatal brain slices caused reduced AMPA receptor levels on medium spiny neurons (129). Further TNFα released from microglia led to reduced excitability of medium spiny neurons in the striatum after cocaine administration (130). In these studies, the actions of TNFα are mediated by TNF receptor 1 expressed on neurons, thus varied neurophysiological effects may be dependent on neuron subtype-specific molecular signaling (128). Separate studies showed repeated social defeat-induced microglia-mediated prostaglandin release that attenuated neuronal responses in the ventral tegmental area (VTA). The reduced firing of VTA neurons following repeated social defeat increased social avoidance, indicating that microglia-mediated modulation of this mesolimbic neuronal pathway contributed to the development of depressive-like behavior (131). These findings provide compelling evidence that microglia produce soluble factors eliciting brain region-specific neuronal responses that elicit cognitive and behavioral consequences.

Other studies show that contact-dependent neuron–microglia interactions are critical modulators of neuronal and behavioral responses to stress. In particular, recent work showed that microglia-mediated elimination of synaptic elements may contribute to stress-induced synaptic plasticity deficits in the hippocampus. For instance, 14 days of chronic unpredictable stress increased the presence of dendritic and synaptic elements in the processes of microglia in the CA1 of the hippocampus. The increased presence of neuronal elements in microglia was associated with reduced sucrose preference and impaired long-term potentiation. Moreover, mice lacking CX_3_CR1 were resilient to CUS, which corresponded with reduced microglia phagocytosis of neuronal elements, attenuated LTP deficits, and normalized sucrose preference (132). It is important to reiterate that mice lacking CX_3_CR1 have delayed neurodevelopment and display baseline social interaction deficits (43, 44), which may contribute to observed stress resilience. It is unclear if microglia expression of CX_3_CR1 is necessary for stress-induced activation, but other studies provide evidence that neuronal CX_3_CL1 activated microglia, which led to the modulation of synaptic strength. For instance, CX_3_CL1 binding to CX_3_CR1 on microglia caused increased IL-1β release and downstream molecular mechanisms that altered synaptic plasticity (133). Further it is important to consider that microglia lacking CX_3_CR1 may be diverted away from homeostatic functions, such as clearance of neural progenitors in the hippocampus, which may exacerbate stress-induced neuropathology (134, 135). These studies demonstrate that microglia can shape neuronal responses to stress through contact-dependent mechanisms, contributing directly to the development of depressive-like behaviors.

The mechanisms that govern microglia-mediated synapse interactions during stress exposure have not been extensively studied; however, as noted microglia are drawn to synapses in an activity-dependent manner. This is pertinent as repeated stress exposure is known to increase neuronal activity in several corticolimbic brain regions, and these neuronal networks are dysregulated in mental health disorders (87, 89). In this context, stress-associated neuronal hyperactivity in specific brain regions may elicit microglia-mediated disruption of synaptic connections with unintended consequences. For instance, microglia disruption of inhibitory synapses on pyramidal neurons may lead to aberrant neuronal activity and extrasynaptic glutamate neurotransmission (136). The potential microglia-mediated exclusion of inhibitory synapses may have consequences on the integrity of interneurons as well. Indeed, recent work indicates that cortical interneurons may be more susceptible to stress-induced dystrophy (85). It is unclear if interneurons are susceptible to stress-induced parainflammation, but deep sequencing techniques in neuron subtype-specific populations may lend insight (137). Further studies will need to expound on these findings to determine how microglia facilitate or disrupt neuroplasticity in stress-responsive brain regions.

In all, microglia have dynamic brain region-specific functions that may contribute to divergent neuroplasticity effects underlying stress-induced anxiety- and depressive-like behavior (5, 13). As the roles of microglia expand, it will be important to characterize brain region-specific phenotypes (28). These studies will undoubtedly reveal unique microglia properties that can be targeted to support adaptive neuronal responses and mental health.

## Summary – Mental Health Disorders as Mild Neurological or Neurodegenerative Disease

Neurons and microglia utilize bidirectional interactions to shape form and function of both cell types. In models of stress-induced mental health disorders, concomitant alterations in neuroplasticity and microglia function are reported, reflecting disruptions in neuron–microglia interactions. Based on these characteristics stress-induced microglia activation resembles an immune state termed parainflammation, which is aimed to restore neuronal homeostasis. It is possible that stress exposure elicits modest inflammatory responses that divert microglia from their supportive functions, leading to neuroplasticity deficits underlying anxiety- and depressive-like behaviors. Stress-induced microglia dysregulation may also contribute to neurological complications as clinical evidence shows that individuals with prior history of mental health disorders have increased risk for dementia or neurodegenerative disease (138, 139). Thus, impaired neuron–microglia interactions may link psychological stress exposure and mental health disorders with aging and neurodegenerative disease (15, 140). This is plausible as synapse loss is a common pathophysiological feature observed in depressed individuals and early stages of neurodegenerative diseases (141, 142). Further studies should be conducted to determine if impaired neuron–microglia interactions contribute to the link between, psychological stress, mental health, and neurodegenerative disease. In addition, recent work indicates that sex-dependent differences in microglia function exist and these likely have implications for the pathophysiology of mental health and cognitive disorders as well (143–145). In the end, pharmacological or molecular pathways that engage or promote the adaptive and neurotrophic functions of microglia may provide therapeutic benefits for mental health and neurological disorders.

## Author Contributions

ESW wrote and edited this manuscript.

## Conflict of Interest Statement

The author declares that the research was conducted in the absence of any commercial or financial relationships that could be construed as a potential conflict of interest.
